# Incidence of Symptomatic Submucous Cleft Palate in the Netherlands: A Retrospective Cohort Study Over a Period of 22 Years

**DOI:** 10.1177/1055665620977760

**Published:** 2020-12-03

**Authors:** Johannes A. Smit, Puck P. Mulder, Feike de Graaf, Bernadette S. de Bakker, Corstiaan C. Breugem

**Affiliations:** 1 Department of Plastic Surgery, Amsterdam University Medical Center, Emma Children’s Hospital, Amsterdam, the Netherlands; 2 Department of Medical Biology, Section Clinical Anatomy & Embryology, Amsterdam UMC, University of Amsterdam, Amsterdam, the Netherlands

**Keywords:** bifid uvula, cleft palate, orofacial clefts, submucosal, submucosal cleft palate

## Abstract

**Objective:**

To analyze the incidence of submucous cleft palate (SMCP) in a large national database and raise awareness among referring providers: pediatricians, speech pathologists, and dentists to minimize delay in diagnosis.

**Design:**

Retrospective cohort study.

**Setting:**

Tertiary setting.

**Patients:**

Patients were extracted from the “Dutch Association for Cleft and Craniofacial Anomalies” database. A total of 6916 patients were included from 1997 until 2018 and divided into 2 groups (ie, SMCP versus cleft palate [CP]). Patients born before 1997 and adopted patients were excluded.

**Interventions:**

Clefts were classified as either hard of soft palatal involvement based on anatomical landmarks at first consultation.

**Main Outcome Measures:**

Primary outcomes were the patient characteristics in both groups (ie, gender, birth weight, gestational age, and additional anomalies). Secondary outcome was the time of diagnosis among subgroups.

**Results:**

In total, 532 patients were diagnosed with SMCP (7.7%). Birth weight, gestational age, and additional anomalies did not differ between subgroups, but there were more males in the SMCP group (*P* < .001). The median age of diagnosis of the SMCP group was significantly higher than of the CP group (987 vs 27 days; *P* < .001). Over the course of 22 years, the time of diagnosis for SMCP did not decrease.

**Conclusion:**

Submucous cleft palate represents <10% of the Dutch cleft population and 19.4% of all CP. Time of diagnosis for SMCP is significantly longer when compared with time of diagnosis of CP, and this has not changed over the study period of 22 years.

## Introduction

Submucous cleft palate (SMCP) is a rare subtype of cleft palate (CP). Although numerous authors describe the incidence of CP, only a few small studies have been conducted on SMCP. Submucous cleft palate was first described in 1825 by Roux (1825). All subtypes are characterized by muscular diastasis of the soft palate with an intact oral and nasal mucosal lining and various different combinations of anatomical abnormalities (Bluestone et al., 2014). Calnan identified a triad of clinical criteria enabling clinicians to diagnose SMCP: a bifid uvula, a notch in the posterior end of the hard palate and a zona pellucida in the midline of the soft palate (Calnan, 1954). Patients who have velopharyngeal insufficiency in the absence of Calnans’ triad are classified as occult SMCP (Kaplan, 1975).

Cleft palate with cleft lip (CLP) is almost always diagnosed before the first year of life. However, Hanny et al. demonstrated that 25% of all patients with CP are diagnosed after 12 months of age (Hanny et al., 2016). Because SMCP is more difficult to diagnose, it could be missed during the initial after birth screening and is therefore often diagnosed late, even later than the mucous CP mentioned by Hanny et al. (Rozendaal et al., 2012). Ten Dam et al. found a median age of 3.7 years at time of diagnosis for SMCP (Ten Dam et al., 2013). Moreover, a recent study demonstrated that 79% of patients with SMCP required surgery (Ha et al., 2013). It is therefore crucial to diagnose these children early in life, offer them speech therapy, and if needed perform surgery to minimize adversary effects later in life. Moreover because a delay in diagnosis could eventually result in hearing impairment and delay in speech development (Ten Dam et al., 2013).

The main objective of this current study is to accurately describe the incidence, patient demographics, and time of diagnosis of patients with SMCP registered in a national database. This information could increase awareness among craniofacial specialists and accommodate early diagnosis at the time when adversary effects are still preventable (eg, delay in speech development).

## Methods

This study is a retrospective cohort study that includes all cleft patients registered in a national database (Dutch Society of Orofacial Cleft and Craniofacial Malformations, NVSCA database) between January 1997 and December 2018 (Vermeij-Keers, 2009). Since January 1997, all craniofacial specialists from tertiary cleft surgery hospitals in the Netherlands (n = 14, 8 academic centers, 6 nonacademic hospitals) register patients with clefts via a digital form. The form comprises of 3 sections (ie, general registration, craniofacial abnormalities, and additional anomalies). The goal of the standardized registration is to provide insight in the distribution of patient demographics (of subtypes) of cleft patients in the Netherlands. Registration is performed by a member of the cleft team during the first outpatient consultation visit.

Characteristics, including gender, ethnicity of patients (adopted vs autochthonous), type of clefts (lip, hard/soft palate, nose; mucosal vs submucosal clefts), birth weight, gestational age, and additional anomalies (eg, atrial/ventricular septal defects, apneas, and hypospadias) were collected. Due to a significant percentage of missing data (ie, >50%), adopted patients, and patients born before January 1997 had to be excluded. Furthermore, patients with cleft lip (CL) and CLP were excluded from further analysis due to heterogeneity of study population. In the current study, only symptomatic SMCP patients are included.

A subdivision was made between patients with SMCP versus patients with CP to investigate the incidence of SMCP and patient demographics. A further analysis was done for patients with or without additional anomalies. The time of diagnosis was investigated for the SMCP cohort and compared to the time of diagnosis of CP patients. The definition of time of diagnosis is the total amount of days between the date of birth and date of registration. Statistical analyses were performed using SPSS (Statistical Program for Social Sciences, version 25; SPSS Inc). For continuous data, normal distributions were evaluated by the Shapiro-Wilk test and Kolmogorov-Smirnov test. If a continuous variable was normally distributed, an independent *t* test was used. If a continuous variable was not normally distributed, a Mann-Whitney *U* test was executed to test for differences between groups. A Pearson χ^2^ test was carried out to investigate the association between 2 categorical variables (eg, gender). A Pearson *r* was applied to investigate correlations between 2 variables. A threshold of *P* < .05 was used to determine statistical significance. The STROBE-checklist (Strengthening the Reporting of Observational Studies in Epidemiology) was adhered to in the preparation of this article.

## Results

**Figure 1.**
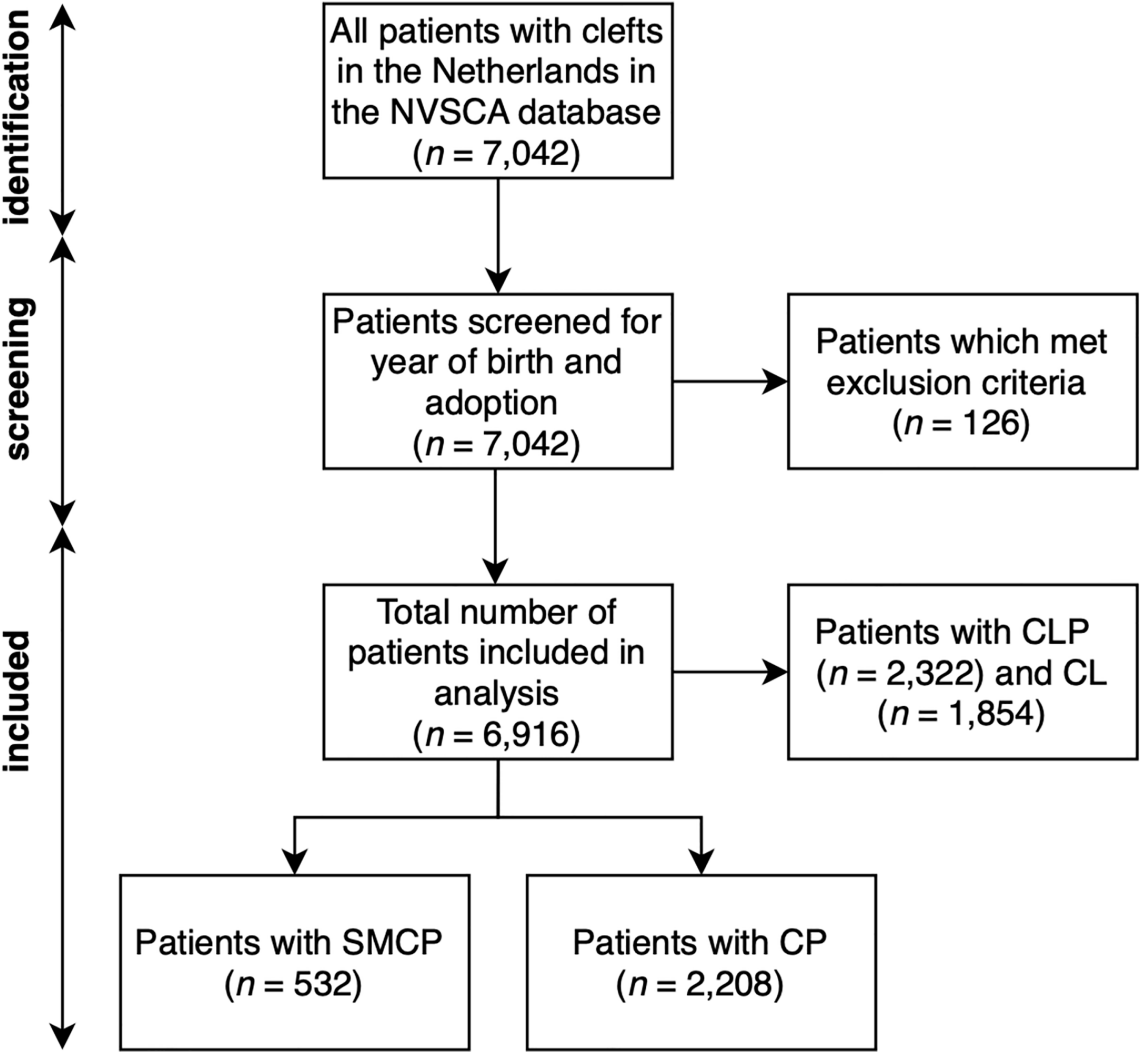
Patient identification and screening.

**Figure 2.**
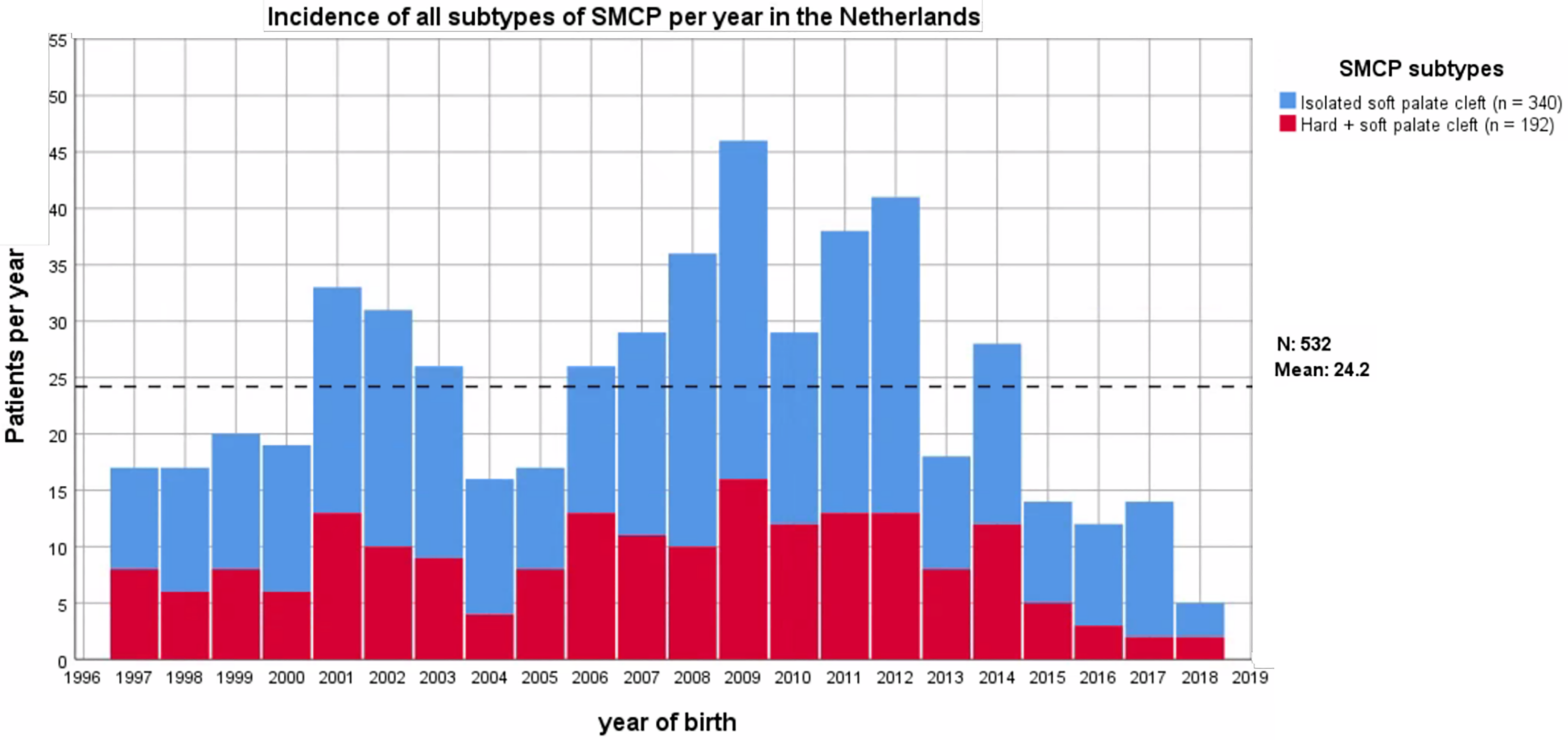
Incidence of subtypes of submucous cleft palate (SMCP) per year in the Netherlands according to the NVSCA database.

In total, 6916 patients with clefts were registered in the Nederlandse Vereniging voor Schisis en Craniofaciale Afwijkingen (NVSCA) database. Of these patients, 532 patients were diagnosed with SMCP; 2208 patients with all other CP (combined to a total of 2740 patients with CP); 2322 patients with CLP and 1854 patients with CL (Figure 1). Of those patients with SMCP, 192 patients were diagnosed with a combination of submucosal cleft of the hard and soft palate (36.1%) and 340 (63.9%) patients were diagnosed with isolated submucosal soft palate cleft (Figure 2). According to the NVSCA database, 314.4 patients per year are born with clefts in the Netherlands. Of these, 24.2 (7.7%) patients are diagnosed with SMCP per year.

Patient characteristics (ie, birth weight, gestational age, gender, and the occurrence of additional anomalies) were analyzed for the SMCP and CP groups (Table 1). The median birth weight of patients with SMCP was 3255 g (≍7 pounds and 2.8 ounces) versus 3314 g (≍7 pounds and 4.9 ounces) for all other CP (*P* = .693). The median gestational age was 39.0 weeks for both the SMCP and CP groups (*P* = .373). Additional anomalies were seen in 132 (24.8%) patients in the SMCP subgroup versus 562 (25.5%) patients in the CP group (*P* = .760). In the SMCP subgroup, 58.3% (n = 310) was male and 41.5% (n = 221) was female (the total of patients does not add up to 100% due to missing data, n = 1). In the CP subgroup, 45.0% (n = 994) was male and 54.8% (n = 1210) was female (missing data in 4 patients). Thus, no differences were found between the SMCP group and CP group concerning birth weight, gestational age, and additional anomalies, but there were significantly more men in the SMCP subgroup versus the CP group (ratio M/F 1.40 vs 0.82; *P* < .001).

**Table 1.** Patient Characteristics.

Characteristics	SMCP (n = 532)	All other CP^a^ (n = 2208)	*P*
Gender^b^ M/F ratio	1.40	0.82	<.001
Male	310 (58.3%)	994 (45.0%)	
Female	221 (41.5%)	1210 (54.8%)	
Birth weight (g.)	3255 (±876)	3314 (±812)	.693
Lbs. + oz. (±oz.)	7 + 2.8 (±30.9)	7 + 4.9 (±28.6)	
Gestational age (weeks)	39.0 (±2.0)	39.0 (±2.0)	.373
Other structural anomalies	132 (24.8%)	562 (25.5%)	.760

Abbreviations: CP, cleft palate; SMCP, submucosal cleft palate.

^a^ Total CP = SMCP + all other CP.

^b^ Note that the total of patients for gender in subgroups does not add up to 100% due to missing data (n = 5).

Additional anomalies were seen in 24.8% in the SMCP subgroup and in 25.5% of the CP group (*P* = .760). To investigate the presence of additional anomalies within different tracts more accurately, SMCP was compared to all other patients with CP specified on all 10 tracts. A list of anomalies with frequencies among patients with SMCP and all other CP is provided (Table 2). Common anomalies seen in patients with SMCP were atrial/ventricular septal defects (n = 23; 4.3%), hypospadias (n = 6; 1.1%) and apneas (n = 4; 0.8%).

**Table 2.** Other Structural Anomalies in SMCP Versus All Other Patients With CP.

Affected tract	SMCP (n = 532)	All other CP (n = 2208)
None	400 (75.2%)	1,646 (80.4%)
Circulatory tract	45 (8.5%)	134 (6.1%)
Respiratory tract	21 (3.9%)	208 (9.4%)
Central nervous system	21 (3.9%)	44 (2.0%)
Urogenital tract	12 (2.3%)	30 (1.4%)
Upper extremity	11 (2.1%)	33 (1.5%)
Digestive tract	11 (2.1%)	42 (1.9%)
Lower extremity	3 (0.6%)	26 (1.2%)
Thorax	3 (0.6%)	19 (0.9%)
Vertebral column	3 (0.6%)	13 (0.6%)
Skin	2 (0.4%)	13 (0.6%)

Abbreviations: CP, cleft palate; SMCP, submucosal cleft palate.

**Figure 3.**
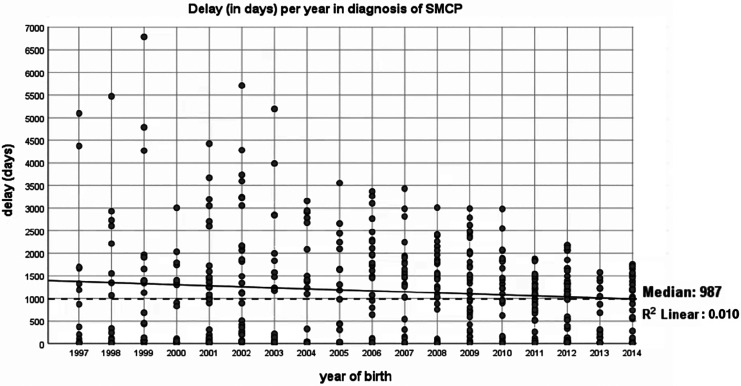
Time of diagnosis of submucous cleft palate (SMCP; in days) per year.

Finally, the time of diagnosis was analyzed per registration year. This time was defined as the total number of days between the date of birth and the date of registration. The median time of diagnosis for all patients with CP was 27 days (ie, slightly less than a month), with an interquartile range (IQR) of 75 days. The median time of diagnosis of SMCP was significantly longer: 987 days (ie, 2 years, 8 months, and 13 days), with an IQR of 1587 days. The time of diagnosis is significantly longer for the SMCP compared to the CP group (*P* < .001) and did not decrease over time (*R* ^2^ = 0.010; Figure 3). To minimize bias, we excluded the last 4 years (ie, median + ¼ IQR) of registration to prevent an underestimation in the last years of our study.

## Discussion

This study demonstrates that SMCP is a relatively common subtype of CP in the Netherlands. In this study, 19.4% (532/2740) of all patients with CP were diagnosed with SMCP. Furthermore, the current study confirms that the time of diagnosis for SMCP remains long, at the age of approximately 3 years.

Several discrete discrepancies were found with recent literature. Discrepancies between incidence and subtypes of SMCP can partly be explained by the fact that it is difficult to accurately classify the anatomy based on a clinic examination during outpatient consultation visits. We found that 7.7% of all patients with clefts were diagnosed with SMCP, while Ten Dam et al. reported that 3.5% of all cleft patients were patients with SMCP (Ten Dam et al., 2013). Although both studies focused on a Dutch population, the difference could be attributed to a smaller study sample: 28 SMCP of 800 total cleft patients in the Ten Dam study versus 524 SMCP of 6916 cleft patients in the current study. Other studies conducted earlier focused on school children for the calculation of the incidence of SMCP (Stewart et al., 1971; Bagatin, 1985). Stewart et al. focused on >10 000 school children and found a total of 9 patients with SMCP, which resulted in an incidence of 1:1200 (Stewart et al., 1971). Bagatin conducted similar research on 9720 Zagrebian school children and found 5 patients with SMCP, which resulted in an incidence of 1:1944 (Bagatin, 1985). Differences in reported incidences could partly be explained by variances in genes accountable for clefting among different study populations (Reiter et al., 2012). Moreover, Stewart et al. and Bagatin investigated asymptomatic children, where in the current study symptomatic children were examined (ie, solely cleft patients were registered). Compared to all live births in the Netherlands from 1997 to 2018 (ie, 185 913 on average per year), the national prevalence of SMCP is 1:7688 (Centraal Bureau voor Statistiek, 2013). A general overview of incidences of SMCP among different studies is provided (Table 3). Differences between studies are mainly dependent on study population and study size.

**Table 3.** Incidence of SMCP in Literature.

Study	Total of SMCP	Study population	SMCP as percentage of all clefts studied	Incidence of SMCP in total population
Current study	532	6916 patients (SMCP + CLP + CP + CL)	7.6%	1:7688^a^
Ten Dam et al. (2013)	28	800 patients (SMCP + CLP + CP + CL)	3.5%	–
Velasco et al. (1988)	1	6000 school children	–	1:6000
Bagatin (1985)	5	9720 school children	–	1:1944
Shprintzen et al. (1985)	19	25 patients with bifid uvulae	–	–
Kono et al. (1981)	33	478 patients (CP)	6.9%^b^	–
Weatherley-White et al. (1972)	61	10 836 school children	–	1:1200
Stewart et al. (1971)	9	>10 000 school children	–	1:1200

Abbreviations: CL, cleft lip; CLP, cleft lip and palate; CP, cleft palate; SMCP, submucous cleft palate.

^a^ Based on the average live births per year according to the Centraal Bureau voor Statistiek (2013) “Central Office of Statistics of the Netherlands.”

^b^ Kono et al did not study patients with CL and CLP.

Gender distribution among patients with SMCP was similar to other studies (Park et al., 2002; Ten Dam et al., 2013). In the current study, 58.3% of patients with SMCP was male and 41.5% was female. In the cohort studied by Ten Dam et al., 61% and 39% of the 28 patients with SMCP were male and female, respectively (Ten Dam et al., 2013). In another study, 54.3% and 45.7% of the 46 patients with SMCP were male and female, respectively (Park et al., 2002). No previous studies among patients with SMCP were conducted on both birth weight and gestational age. Compared to the average birth weight from 2001 to 2013, as reported by Statistics Netherlands, patients with SMCP in this study weighed less than the national average (3255 g ≍ 7 pounds and 2.8 ounces for patients with SMCP compared to 3433 g ≍ 7 pounds and 9.1 ounces for all newborns in the Netherlands) (Centraal Bureau voor Statistiek, 2013). Unfortunately, no average gestational age of Dutch newborns is available. A previous study by Wyszynski et al. also found that patients with orofacial clefts are more at risk to have a lower birth weight but did not see increased risk of premature birth (Wyszynski et al., 2003). Further research needs to be conducted to compare our findings to other study populations with regard to birth weight and gestational age.

The median age of diagnosis for SMCP was found to be at 987 days (ie, 2 years, 8 months, and 13 days). This is earlier than previously reported (3.9-4.9 years) (Reiter et al., 2011; Ten Dam et al., 2013), which could be explained by a small sample size (ie, 28 patients with SMCP) (Ten Dam et al., 2013) and the use of a more dated cohort (start cohort in 1981 versus 1997) (Reiter et al., 2011). The long time of diagnosis means that patients with SMCP remain undiagnosed for a long time, which could impair speech development and cause other adversary effects later on (Ten Dam et al., 2013). In addition, in a recent study by Ha et al., 79% of SMCP patients required surgery (eg, for speech improvement) (Ha et al., 2013). It is therefore crucial to diagnose these children early in life, offer them speech therapy and perform surgery, if necessary, to minimize adversary effects later on. Parents of patients seek medical help in advanced stages of SMCP, often when speech therapists cannot further improve speech (Ten Dam et al., 2013). This study did not investigate why patients with a SMCP were send to the physicians, but a recent study by Hanny et al. has demonstrated that these patients often have feeding problems as babies and subsequently speech problems needing speech therapy later in life. Although midwives/pediatricians should investigate the mouth/palate after birth, pediatricians should be aware that children with feeding problems and possible nasal regurgitation could have a submucous cleft. Moreover, if patients need speech therapy because of nasal speech, speech pathologists should also be aware of a possible SMCP. Unfortunately, we found that the time of diagnosis did not decrease throughout the years of registration. In the United Kingdom, it has been advocated that midwives, pediatricians, and gynecologists should not only look at the palate but also perform digital examinations to diagnose SMCP more accurately and earlier (Royal College of Paediatrics and Child Health, 2018). In the Netherlands, midwives are only obliged to look and not feel for a possible submucous cleft.

A large sample size of patients with SMCP was analyzed from multiple hospitals, and only 3 exclusion criteria (ie, adopted patients with clefts, patients born before 1997 and patients with CL and CLP) were applied. Consequently, this study has a high external validation. A possible drawback of the study is the classification used in the Netherlands that focused solely on the hard and soft palate, compared to the more elaborate classifications used by Khan et al. (hard palate anterior of the incisive foramen affected; palatine muscles of the maxillary bone affected; palatine muscles of the palatine bone affected; soft palate affected) (Khan et al., 2013), or the even more elaborate scoring system used by Sommerlad et al., who created a scoring system from 1 to 9, taking in account the hard palate, the uvula, and muscles of the palate (Sommerlad et al., 2004). Another possible disadvantage is the way in which additional anomalies were registered. The registration of additional anomalies is a snapshot at the first outpatient consultation. Thus, additional anomalies that developed later in life were not reported (eg, undiagnosed heart/digestive tract anomalies). Due to the retrospective design of this study, we were not able to investigate which percentage of patients with SMCP needed speech surgery. More research is needed to address this specific question.

## Conclusion

This study demonstrates that SMCP is a rare subtype of cleft in the Netherlands. Submucous cleft palate represents 7% of all clefts seen in the Netherlands and 19.4% of all CP in the Netherlands, with an incidence of 1:7688 live births over the studied period of 22 years. Time of diagnosis is evident and remains long over the years compared to time of diagnosis of other CP. Moreover, the results of this study underline the fact that the SMCP subpopulation does not differ from other subtypes of clefts with regard to birth weight, gestational age, and additional anomalies, which make an elaborated physical examination (look and palpate the oral cavity) even more essential in early diagnosis. Improvement in knowledge on SMCP among pediatricians, midwives, and speech pathologists could raise awareness, which expedites diagnosis and could prevent potential adversary effects later in life.
